# Exogenous emulsifiers and multi-enzyme combination improves growth performance of the young broiler chickens fed low energy diets containing vegetable oil

**DOI:** 10.5713/ab.22.0024

**Published:** 2022-04-22

**Authors:** Samiru Sudharaka Wickramasuriya, Shemil Priyan Macelline, Eunjoo Kim, Taeg Kyun Shin, Hyun Min Cho, Dinesh D. Jayasena, Jung Min Heo

**Affiliations:** 1 Department of Animal Science and Biotechnology, Chungnam National University, Daejeon 34134, Korea; 2 School of Life and Environmental Sciences, Faculty of Science, University of Sydney, Sydney 2006, Australia; 3 School of Environmental and Rural Science, University of New England, Armidale, NSW, 2351, Australia; 4 Department of Animal Science, Uva Wellassa University, Badulla 90000, Sri Lanka

**Keywords:** Broiler, Emulsifier, Growth Performance, Multi-enzyme, Nutrient Digestibility

## Abstract

**Objective:**

The present study examined the effects of exogenous emulsifiers and multi-enzyme supplementation into a low energy density diet on growth performance, visceral organ parameters, blood metabolites, ileal morphology, and nutrient digestibility in broiler chickens from hatch to 21 days.

**Methods:**

One hundred and sixty-eight one-day-old Ross 308 broiler chickens were allocated in a completely randomized design to 24 pens and each pen was assigned to one of four dietary treatments to give six replications with seven chickens in a cage. Dietary treatments were: i) positive control with standard energy level (PC); ii) negative control with 100 kcal/kg lower energy of the standard level (NC); iii) NC diet supplemented 0.05% calcium stearoyl-2 lactylate as an emulsifier (NC+E); and iv) NC diet supplemented with both 0.05% calcium stearoyl-2 lactylate and 0.05% multi-enzyme (NC+E+M). Corn and soybean meal-based control diets containing vegetable oil were formulated to meet the Ross 308 nutrition specification. Chickens were fed *ad-libitum* with the treatment diets and sampling was conducted on day 21.

**Results:**

Our results revealed that emulsifier and multi-enzyme supplementation into NC diets improved (p<0.05) feed efficiency of the broiler chickens compared to the broiler chickens fed NC diets from hatch to 21 days. Supplementation of emulsifier and multi-enzyme into NC diet improved (p<0.05) nutrient digestibility of the broiler chickens. However, emulsifier and multi-enzymesupplementation into diet did not influence (p>0.05) visceral organ weight, blood metabolites, and intestinal morphology in broiler chickens fed NC diets.

**Conclusion:**

Supplementation of emulsifier and multi-enzyme in the NC diet would support improving growth performance in young broiler chickens with improved feed efficiency and increased nutrient digestibility thereby curtailing the negative impact of energy reduction in the diets.

## INTRODUCTION

The least-cost feed formulation is important to attain conducive profit margins in the broiler industry while maintaining ideal growth performance in broiler chickens. Dietary such as formulating low-density nutrient diets with alternative low-cost feed ingredients would reduce the diet costs, and thereafter supplementation of suitable (e.g., exogenous enzymes, bile acid, and emulsifiers) could reinforce the poor nutrient availability and digestibility of such diets. Broiler diets’ supplementation with exogenous enzymes, such as carbohydrases, phytases, proteases, and lipases [1,2], and emulsifier [3] improve nutrient utilization of diets with alternative feed ingredients (e.g., cereals, cereal by-products, fat, and oils), and consequently enhance growth performance in broiler chickens.

Dietary fat and oil were greatly overlooked as substantial energy sources in broiler diets, despite their higher energy content and relatively lower cost than cereal-based energy sources [3]. In addition, provision of essential fatty acids, supporting fat-soluble vitamin absorptions, and improving feed texture quality (pellet durability index) are added advantages of fats and oil in poultry feed. Dietary lipid sources need to be emulsified by emulsifiers in the digestion process to support efficient digestion and absorption. However, it was reported that young broilers have lower endogenous emulsifier production, therefore, fat digestibility in the premature gut is less likely to be efficient for optimum energy uptake [4]. Therefore, to improve fat digestibility in young broiler chickens, exogenous emulsifier supplementation is being researched.

Similar to emulsifiers, the importance of carbohydrase in broiler diets was tested as these enzymes can enhance the nutritional value of cereals and cereal by-products that are rich in non-starch polysaccharides (NSP) [5,6]. The exogenous carbohydrase plays a variety of roles in broiler diets such as degradation of NSP in cell wall matrixes, exposes encapsulated nutrients to digestive enzymes, improves nutrient retention, and modulates the gut viscosity and passage rate [5].

Beyond the single enzyme application, many studies have been conducted to evaluate the broiler responses to various enzyme combinations. Combinations, like carbohydrase and proteases; carbohydrase and phytase; and carbohydrase, protease, and phytase; have been reported for broiler chickens [5,7,8]. It is evident that supplementation of emulsifiers with a multi-enzyme blend in low nutrient-density diets partially improves the growth performance and relative organ weight in broiler chickens [9]. Wang et al [10] reported that supplementation of an emulsifier-carbohydrase combination in a low-density energy diet partially improved the growth performance of broiler chickens.

However, limited research information exists on the effect of exogenous emulsifiers and multi-enzyme combinations on the growth performance of broiler chickens. Having more studies pertaining to emulsifier and multi-enzyme and its knowledge is timely needed to the field of broiler nutrition. To our knowledge, the calcium stearoyl-2 lactylate together with multi-enzyme combination has not been reported previously in broiler chickens. Consequently, the objective of the present study was to evaluate the effect of exogenous emulsifiers and multi-enzyme combinations on growth performance, blood metabolites, viscera organ weights, ileal morphology, and nutrient digestibility in broiler chickens fed corn and soybean meal-based diet containing vegetable oil as the dietary fat source.

## MATERIALS AND METHODS

The experiment protocol for the present study was approved by the Animal ethics committee of the Chungnam National University (Protocol No. CNU - 00863).

### Chickens, diets, and management

A total of 168 broiler chickens (Ross 308; one-day-old) were arranged in a completely randomized design and allotted to one of four dietary treatments. Each treatment consisted of six replicate pens (seven chickens per pen) with even distribution of initial body weights (53.13±0.68 g). Chickens were raised in wired-floor pens (0.85×0.55×0.35 m^3^) under temperature-controlled environment conditions and all the management practices were followed Ross 308 broiler management guidelines [11] from 1 to 21 days of age.

Corn and soybean-meal-based diets with two different energy densities (PC, standard energy density vs NC, 100 kcal/kg energy-deficient to PC) were formulated (Table 1), to meet the Ross broiler 308 broiler nutrient specification [12]. Soybean oil served as an energy source for both diets. To made up the rest of the two dietary treatments, NC diets were supplemented with either CSL (0.05% calcium stearoyl-2 Lactylate; SNH Biotech Co., Ltd. Daejeon, Korea) or CSL with multi-enzyme (0.05% Superzyme-CS; Canadian Bio-Systems Inc., Calgary, AB, Canada). The added multi-enzyme contained 1,200 U of xylanase, 150 U of glucanase, 700 U of invertase, 5,000 U of protease, 500 U of cellulase, 12,000 U of amylase, and 60 U of mannanase as calculated enzyme activities per kilogram of diet. As an inert marker, 0.3% of Cr_2_O_3_ (>99.9%, Sigma-Aldrich, St. Louis, MO, USA) was added into all treatment diets to determine the ileal nutrient digestibility of the broiler chickens. Chickens were offered the experimental diets on an *ad-libitum* basis and had free access to fresh clean drinking water via nipple drinkers throughout the experiment period.

### Growth performance

On days 1 and 21 pen basis body weights, and feed intake were recorded. Feed intake was measured as the feed disappearance of the individual feeder. Based on the measured body weight and feed intake, pen basis average daily gain (ADG), average daily feed intake, and feed conversion ratio (FCR) were calculated. All feed intake and feed conversion data were corrected for the mortalities.

### Sample collection

At the end of the experiment on day 21, Six chickens per treatment (one from each replicate) were randomly selected, recorded the live body weight, and collected the blood samples that were drawn from the Jugular vein into BD Vacutainer SST tubes containing polymer gel for serum separation (BD Biosciences, Franklin Lakes, NJ, USA). Thereafter, chickens were euthanized by cervical dislocation for sample collection.

The gastrointestinal tract was exposed to excised the ileum samples from each sacrificed bird. The ileum was defined as the segment of the small intestine that extended from Meckel’s diverticulum to the ileocecal junction. A 3 cm piece of middle ileum was removed and flushed with ice-cold phosphate-buffered saline at pH 7.4. The samples were placed into plastic containers containing 10% formaldehyde for fixation and stored until analyzed the intestinal morphology. The remaining ileum segment was gently flushed into a labeled sample container to collect the remaining digesta for nutrient digestibility analysis. Collected digesta samples were stored −20°C for later analysis. Following the separation of ileal samples, liver, spleen, and gizzard were removed and weighed separately. Contents and the excess fat were removed manually, blotted dry, and recorded the organ weights. The liver, spleen, and gizzard weights were expressed as proportions of the live body weight of the respective chickens.

### Sample analyses

Collected and clotted blood samples were then centrifuged (LABOGENE 1248R; Gyrozen Co., Ltd., Daejeon, Korea) to separate serum at 3,000×g for 10 min at 4°C and stored at −80°C (UniFreez U 400; DAIHAN Scientific Co., Ltd, Gangwon, Korea.) until analysis was performed. Afterward, serum cholesterol, glucose, lipase, and triglyceride levels were analyzed using HITACHI 7180 chemistry analyzer (HITACHI, Tokyo, Japan).

Collected ileal tissue samples were processed to make microscopic slides as described by Wickramasuriya et al [13]. Briefly, ring-shaped lengths of ileum samples were resected, dehydrated, and embedded in paraffin wax. From each of these, six transverse sections (4 to 6 μm) were excised, stained with hematoxylin and eosin, and mounted on glass slides. The 10 morphological measurements from each slide were taken from well-oriented villi and their associated crypts using NIS-Elements Viewer software (Version: 4.20; NIS Elements, Nikon, Melville, NY, USA) with an inverted microscope (Eclipse TE2000; Nikon Instruments Inc., USA) using a calibrated eyepiece graticule.

Dry matter, crude protein, and gross energy were measured as the method described previously [14]. Ileal digestibility of nutrients was calculated based on the following equation.


$$Digestibility,\%=100\;\left[1-\left(\frac{{Cr}_{feed}}{{Cr}_{Digesta}}\right)\times \left(\frac{{Nut}_{Digesta}}{{Nut}_{feed}}\right)\right]$$

Where Cr and Nut are the chromium dioxide and test nutrient, respectively.

### Statistical analyses

Data were analyzed as a completely randomized design using SPSS software (Version 21; IBM SPSS 2012). One-way analysis of variance in the general linear model procedure was performed to determine the significates of the mean. The pen was used as the experimental unit for all growth performance measurements and selected individual chickens were considered the experimental unit for blood parameters, viscera organ weights, ileal morphology, and nutrient digestibility. Mean differences observed in the treatment were considered significant at p<0.05. When treatment effects were significant (p<0.05), means were separated using Duncan multiple range test procedures of SPSS software (Version 24; IBM SPSS 2016).

## RESULTS

The effects of dietary treatments on growth performance in broiler chickens are shown in Table 2, where PC diet outperformed NC diet on day 21 (p<0.05). Emulsifier and emulsifier plus multi-enzyme supplementation to NC diet restored the ADG in broiler chicken, where PC diet and NC+E or NC+ E+M diets had significantly similar ADGs at 21 days post-hatch (p>0.05). However, only emulsifier supplementation to NC diet did not support FCR, while a combination of emulsifier and multi-enzyme supplementation to NC diet improved FCR by 9.86%, compared to NC diet (1.38 vs 1.53 g/g), which is significantly similar to PC diet.

The impact of different dietary treatments on relative visceral organ weights is shown in Table 3. The relative weights of the liver, spleen, and gizzard were not influenced by the dietary treatments (p>0.05).

The blood metabolic responses to dietary treatments are shown in Table 4, where any of the considered metabolites (cholesterol, glucose, lipase, and triglyceride) were not influenced by the dietary treatments (p>0.05). However, dietary treatments showed a numerical trend (p = 0.056) of serum glucose concentrations such that PC diet had the highest (301.4 mg/dL) and NC+E+M diet had the lowest (274.7 mg/dL) concentrations.

The effect of dietary treatments on intestinal morphology is shown in Table 5. Broilers offered PC and NC diets had similar (p>0.05) villus heights, crypt depth, and villus height to crypt depth ratio in the ileum. Also, either emulsifier or emulsifier plus multi-enzyme supplementation did not influence the ileum morphology in broiler chickens (p>0.05).

Apparent nutrient digestibility in the ileum was influenced by the dietary treatments (p<0.05) as shown in Table 6. The NC diet showed decreased dry matter, crude protein, and energy digestibility by 21.8%, 15.3%, and 26.9%, respectively, than the PC diet. Emulsifier supplementation to NC diet enhanced the nutrient digestibility, as NC+E diet had better dry matter, crude protein, and energy digestibility by 20.1%, 11.5%, and 17.6%, respectively, over NC diet and were not significantly different from that of the PC diet. Moreover, supplementation of emulsifier and multi-enzyme further improved nutrient digestibility, while NC+E+M has comparable responses to PC.

## DISCUSSION

A plethora of studies has been reported on improved growth performance and nutrient digestibility by the addition of exogenous emulsifiers or carbohydrase into broiler diets. Previously we reported different emulsifier’s ability to improve nutrient digestibility and growth performance of the broiler chickens [3,13]. In a recent study [15], lysophospholipid-based bio-emulsifier showed an improved nutrient digestibility and growth performance of broiler chickens. Similarly, Bontempo et al [4] observed beneficial effect of synthetic emulsifier product (consisting of vegetal bi-distilled oleic acid emulsified with ethoxylated castor oil) supplementation into broiler diets on growth performance, lipid metabolism and carcass parameters. Reported emulsifier effects on chickens and comparisons of emulsifying agents were also reviewed by Siyal et al [16]. Similar to emulsifiers, addition of carbohydrase or multi-enzyme combination into broiler diets have been reported to improve growth performance and nutrient digestibility of broiler chickens [2,6,7]. Nevertheless, the synergetic effect of exogenous emulsifiers and carbohydrase or multi-enzyme on broiler chickens is scantly documented, and only a couple of studies have reported the synergetic effect of exogenous emulsifiers and carbohydrase combination in broiler chickens after the year 2015 [10,17]. Improved growth performance and feed efficiency in broiler chickens fed low energy diet supplemented with an emulsifier (sodium stearoyl-2-lactylate) and carbohydrase (a blend of α-galactosidase, galactomannans, xylanase, and β-glucanase) combination has been reported by Wang et al [10]. The effect of xylanase and emulsifier combination in wheat-based diets on gastrointestinal tract microbiota activity and gut morphology in broiler chickens has been investigated by Kubiś et al [17]. Therefore, it would be worth investigating the synergistic effect of exogenous emulsifiers and multi-enzyme combinations on broiler performance as a potential dietary approach for improving nutrient utilization and reducing feed cost in broiler chickens.

Previously, both emulsifier (calcium stearoyl-2 lactylate) and multi-enzyme used in the present study were tested on broiler chickens by Wickramasuriya et al [13] and Wickramasuriya et al [2], respectively. Based on observed results of those studies, we performed this experiment with the putative view of better growth response and improved nutrient digestibility of young broiler chickens.

The inferior growth performance in broilers offered NC diet confirmed that a 100 kcal/kg dietary energy reduction was sufficient to create a sensible adverse effect on the performance of broiler chickens. Identical to present study, some other researchers [18–20] also aimed a sensible growth outcome with 100 kcal/kg dietary energy reduction in broiler chickens. Interestingly, NE+E and NE+E+M diets restored the final body weights and resulted in weight gain in those fed the NC diet, to reach the weight of those fed the PC diet, which indicated the importance of synergetic effects of exogenous emulsifier and multi-enzyme combinations in the present study. However, only the NE+E+ M diet was able to improve FCR of those fed the PC diet in the present study, which indicates that the interactive impact of emulsifier and multi-enzyme is important to obtain better FCR in energy-deficient broiler diets.

Interestingly, the NC+E diet supported 10.7% higher feed intake than the NC+E+M diet (69.17 vs 62.49 g/bird/d), which might be because of energy deficiency in broilers offered NC+E diet therefore, chickens would have to increase feed intake to correct their energy requirements. Dietary energy intake between treatments did not significantly differ in the present study (data not shown, p = 0.145), indicating that chickens are eager to correct their energy requirements by adjusting the feed intake and this rationale was discussed comprehensively by Classen [21]. Therefore, compromised FCR in NC+E diet compared with NC+E+M diet could be derived because of higher feed intake in broilers offered the NC+E diet, which might be associated with their energy demand which relies on the diet. In the present study, the extra 100 kcal/kg energy in the PC diet over the NC diet must be derived from dietary starch which is almost entirely digestible in broiler chickens, and it has been reported that the same emulsifier used in the present study did not improve the crude fat digestibility, as described by Wickramsuriya et al [13]. Therefore, sole supplementation of emulsifiers to low-starch, energy-deficient diets did not compensate for energy utilization and growth performance in broiler chickens. However, it is interesting to inspect the benefit of the multi-enzyme and emulsifier combination effect, where similar outcomes of improved growth performance in broiler chickens fed an energy-deficient diet were reported [10]. Nevertheless, our previous studies [2,6] on the same multi-enzyme supplementation did not show any significant influence on FCR or energy utilization in broiler chickens from hatch to 21 days. Despite that, emulsifier and multi-enzyme combination supplementation significantly improved the energy and crude protein digestibility which would be the main reason for this synergetic effect associated with better growth performance in the present study.

Looking into the diet formulation, lower nutrient digestibility in a low energy density diet is thought to be associated with higher inclusions of wheat bran as it reduces nutrient digestibility in broiler chicken via increased gut viscosity, nutrient encapsulation, and decreased digesta passage rate along the gut [22]. This postulation is further confirmed in our recent study in which no nutrient digestibility differences were observed in lowering the dietary energy level while maintaining the same NSP profile [2]. Findings in the present study are in broad agreement with those by Zhang et al [23], which reported the lower nutrient digestibility in the chickens fed fermented wheat bran substituted diet containing soybean oil. In this study, higher nutrient digestibility in carbohydrase and emulsifier combination may be attributed to the carbohydrase action of degradation of cell wall polysaccharides, facilitating endogenous enzymes to digest more nutrients in the feed matrix. With the agreement of carbohydrase action on nutrient digestibility of broiler diets, Amerah et al [24] reported the addition of xylanase and amylase, and their combination improved the nutrient digestibility of the broiler chickens fed a corn-soy diet. However, the role of emulsifier in the NE+E+M diet for improved energy utilization is not clear, while NE+E did not significantly improve energy utilization but numerically increased energy digestibility by 18% (66.2% vs 77.9%) than the NE diet. Moreover, as per our previous studies, multi-carbohydrases did not improve energy digestibility in broilers without emulsifiers at day 21 post-hatch. Therefore, increased energy digestibility in the NE+E+M diet may be the result of the additive effect of fat digestion and starch digestion by the emulsifier and multi-enzyme, respectively.

## CONCLUSION

The supplementation of CSL as an emulsifier together with multi-enzyme, synergistically improved growth performance of the broiler chickens fed low energy vegetable-oil based diet containing wheat bran, as a result of improved nutrient digestibility. Therefore, emulsifier and multi-enzymee combinations could be a promising strategy to reduce dietary energy density while maintaining the growth performance in broiler chickens from 1 to 21 days post-hatch.

## Figures and Tables

**Table 1:** Ingredient and calculated nutrient composition of experimental diets (%, as-fed basis)

Items	Positive control	Negative control
Ingredient		
Corn	48.00	43.28
Wheat bran	4.17	9.79
Soybean meal, 44%	39.60	38.70
Vegetable oil^1)^	4.00	4.00
Limestone	1.50	1.50
Monocalcium phosphate	1.70	1.70
Salt	0.35	0.35
Vitamin-mineral premix^2)^	0.30	0.30
Lysine-HCl	0.10	0.10
DL-Methionine	0.28	0.28
Nutrient specifications^3)^		
Metabolizable energy (kcal/kg)	3,050	2,950
Crude protein (%)	22.28	22.32
Crude fat (%)	6.29	6.30
Calcium (%)	1.09	1.10
Available phosphorous (%)	0.49	0.50
Total lysine (%)	1.36	1.36
Total methionine (%)	0.62	0.62
Total methionine+cysteine (%)	1.00	1.01
Total threonine (%)	0.87	0.86
Total tryptophan (%)	0.28	0.28
Total valine (%)	1.03	1.03
Total arginine (%)	1.48	1.49
Analyzed values		
Gross energy (kcal/kg)	4,173	4,147
Crude protein (%)	22.92	22.84

1) Soybean oil.

2) Supplied per kilogram of total diets: Fe (FeSO_4_·H_2_O), 80 mg; Zn (ZnSO_4_·H_2_O), 80 mg; Mn (MnSO_4_·H_2_O) 80 mg; Co (CoSO_4_·H_2_O) 0.5 mg; Cu (CuSO_4_·H_2_O) 10 mg; Se (Na_2_SeO_3_) 0.2 mg; I, (Ca(IO_3_)·2H_2_O) 0.9 mg; vitamin A, 24,000 IU; vitamin D_3_, 6,000 IU; vitamin E, 30 IU; vitamin K, 4 mg; thiamin, 4 mg; riboflavin, 12 mg; pyridoxine, 4 mg; folacine, 2 mg; biotin, 0.03 mg; vitamin B_8_, 0.06 mg; niacin, 90 mg; pantothenic acid, 30 mg.

3) The values were calculated according to the values of feedstuffs in NRC (1994).

**Table 2:** Effects of dietary energy levels, emulsifier, and multi-enzyme supplementation on growth performance of broiler chickens from 1 to 21 days of age

Item	Treatment^1)^				SEM	p-value

	NC	PC	NC+E	NC+E+M		
Final body weight (g)	965.96^a^	1,054.67^b^	1,011.26^ab^	1,016.74^ab^	10.629	0.020
ADG (g/bird/d)	43.48^a^	47.70^b^	45.62^ab^	45.47^ab^	0.500	0.018
ADFI (g/bird/d)	66.30	65.84	69.17	62.49	0.886	0.069
FCR (g/g per bird)	1.53^b^	1.39^a^	1.52^b^	1.38^a^	0.025	0.032

SEM, pooled standard error of mean; ADG, average daily gain; ADFI, average daily feed intake; FCR, feed conversion ratio.

1) NC, low energy control diet; PC, standard energy control diet; NC+E (NC+0.05% emulsifier), NC+E+M (NC+0.05% emulsifier+0.05% multi-enzyme).

a,b Means in the same row with different superscripts differ (p<0.05).

**Table 3:** Effects of dietary energy levels, emulsifier and multi-enzyme supplementation on relative visceral organ weights of broiler chickens on 21 days of age

Item (%)	Treatment^1)^				SEM	p-value

	NC	PC	NC+E	NC+E+M		
Liver	2.56	2.58	2.86	2.78	0.055	0.130
Spleen	0.08	0.08	0.09	0.09	0.006	0.935
Gizzard	1.41	1.57	1.66	1.55	0.045	0.278

SEM, pooled standard error of mean.

1) NC, low energy control diet; PC, standard energy control diet; NC+E (NC+0.05% emulsifier), NC+E+M (NC+0.05% emulsifier+0.05% multi-enzyme).

**Table 4:** Effects of dietary energy levels, emulsifier and multi-enzyme supplementation on blood serum metabolites of broiler chickens on 21 days of age

Item	Treatment^1)^				SEM	p-value

	NC	PC	NC+E	NC+E+M		
Cholesterol (mg/dL)	143.58	140.90	136.68	140.40	3.091	0.903
Glucose (mg/dL)	292.90	301.40	275.05	274.71	4.185	0.056
Lipase (U/L)	10.77	11.85	12.88	12.68	0.425	0.290
Triglyceride (mg/dL)	61.72	83.80	72.78	74.38	4.504	0.408

SEM, pooled standard error of mean.

1) NC, low energy control diet; PC, standard energy control diet; NC+E (NC+0.05% emulsifier), NC+E+M (NC+0.05% emulsifier+0.05% multi-enzyme).

**Table 5:** Effects of dietary energy levels, emulsifier and multi-enzyme supplementation on the ileum morphology of broiler chickens on 21 days of age

Item	Treatment^1)^				SEM	p-value

	NC	PC	NC+E	NC+E+M		
Villus height (V, μm)	529.81	537.16	597.69	495.65	15.824	0.140
Crypt depth (C, μm)	62.63	61.29	51.99	49.31	3.070	0.334
V:C ratio	10.31	9.00	11.85	10.63	0.520	0.296

SEM, pooled standard error of mean.

1) NC, low energy control diet; PC, standard energy control diet; NC+E (NC+0.05% emulsifier), NC+E+M (NC+0.05% emulsifier+0.05% multi-enzyme).

**Table 6:** Effects of dietary energy levels, emulsifier and multi-enzyme supplementation on the ileal digestibility of nutrients of broiler chickens on 21 days of age

Item (%)	Treatment^1)^				SEM	p-value

	NC	PC	NC+E	NC+E+M		
Dry matter	63.49^a^	81.17^b^	76.22^ab^	81.52^b^	2.234	0.005
CP	75.14^a^	88.70^b^	83.79^ab^	87.94^b^	1.682	0.004
Energy	66.23^a^	83.75^b^	77.91^ab^	82.08^b^	2.293	0.019

SEM, pooled standard error of mean.

1) NC, low energy control diet; PC, standard energy control diet; NC+E (NC+0.05% emulsifier), NC+E+M (NC+0.05% emulsifier+0.05% multi-enzyme).

a,b Means in the same row with different superscripts differ (p<0.05).
